# Blood and urine biomarkers of disease progression in IgA nephropathy

**DOI:** 10.1186/s40364-024-00619-4

**Published:** 2024-07-29

**Authors:** Zhi-Yu Duan, Chun Zhang, Xiang-Mei Chen, Guang-Yan Cai

**Affiliations:** Department of Nephrology, State Key Laboratory of Kidney Diseases, Beijing Key Laboratory of Kidney Diseases Research, First Medical Center of Chinese PLA General Hospital, National Clinical Research Center for Kidney Diseases, Beijing, 100853 China

**Keywords:** IgA nephropathy, Progression, End-stage renal disease, Biomarkers, Prediction

## Abstract

The prognosis of patients with IgA nephropathy (IgAN) is variable but overall not good. Almost all patients with IgAN are at risk of developing end-stage renal disease within their expected lifetime. The models presently available for prediction of the risk of progression of IgAN, including the International IgA Nephropathy Prediction Tool, consist of traditional clinical, pathological, and therapeutic indicators. Finding biomarkers to improve the existing risk prediction models or replace pathological indicators is important for clinical practice. Many studies have attempted to identify biomarkers for prediction of progression of IgAN, such as galactose-deficient IgA1, complement, a spectrum of protein biomarkers, non-coding RNA, and shedding cells. This article reviews the biomarkers of progression of IgAN identified in recent years, with a focus on those with clinical value, in particular the combination of multiple biomarkers into a biomarker spectrum. Future research should focus on establishing a model based primarily on biomarkers that can predict progression of IgAN and testing it in various patient cohorts.

## Introduction

IgA nephropathy (IgAN) is the most common type of primary glomerulonephritis worldwide [1] and the type most widely seen on renal biopsy in the Chinese population [2]. The prognosis of IgAN varies greatly, but overall is unfavorable. A recent analysis of the IgAN cohort data from the UK National Registry of Rare Kidney Diseases suggests that 50–60% of patients with IgAN develop end-stage renal disease (ESRD) within 10–15 years, and almost all of these patients are at risk of developing ESRD within their expected lifetime [3]. Risk stratification with early identification of high-risk patients and intervention are important for improvement of the prognosis. The International IgA Nephropathy Prediction Tool (IIGANPT) is a risk prediction model based on large-scale data obtained from multiple centers. Published in 2019, this tool can accurately predict the risk of renal progression (ESRD or a > 50% decrease in estimated glomerular filtration rate [eGFR]) in patients with IgAN within 80 months of renal biopsy [4] and is recommended in the 2021 Kidney Disease: Improving Global Outcomes (KDIGO) guidelines [5]. This model integrates pathological and clinical indicators and the treatment plan. Pathological indicators are an important component of the IIGANPT model, which has been updated to include risk stratification of the prognosis using indicators measured 1–2 years after renal biopsy [6]. However, the IIGANPT model still has some limitations. The need for pathological indicators means that it is not possible to repeatedly evaluate changes in the patient’s condition as needed during the long course of the disease. Pathological data obtained by renal biopsy is currently the “gold standard” for diagnosis and evaluation of the prognosis in most patients with kidney disease, including IgAN. However, renal biopsy is invasive with certain contraindications and a risk of adverse events. Furthermore, patients with IgAN may experience multiple sudden exacerbations and even acute kidney injury (AKI) during the long course of the disease. At this time, pathological information obtained by renal biopsy at onset of the disease cannot be used to evaluate or predict renal progression. Therefore, there is an urgent need to find prognostic biomarkers of IgAN that can be used in clinical practice.

In recent years, “liquid” biopsy methods, including measurement of proteins and DNA methylation, microRNA (miRNA), and long non-coding RNA (lncRNA) and single-cell sequencing in blood or urine, have been used for diagnosis and assessment of changes in kidney diseases. The aims of this review are to evaluate the ability of the blood and urinary biomarkers identified so far to predict progression of IgAN, improve our understanding of methods for assessment of progression of the disease, and accelerate the transition from basic research to clinical practice.

### Clinical risk factors

Multiple models suggest that urinary protein quantification, eGFR, and blood pressure (BP) predict the risk of progression of IgAN [4, 7]. Mean arterial pressure (MAP) was used in the original version of the IIGANPT [4] and continues to be used in the modified version [6]. In a retrospective study involving 2945 patients with IgA nephropathy, patients who had a history of hypertension was good blood pressure control in the first year after diagnosis, it remained a risk factor for IgAN progression [8]. More recent research found that, in addition to high BP or MAP, variability in BP during visits was associated with progression of IgAN [9]. In the same study, a higher standard deviation (SD) of systolic BP after adjustment for both baseline and mean values showed a significant correlation with progression of IgAN. Furthermore, the standard deviation of diastolic BP could not predict disease progression. For the control of BP in patients with IgA nephropathy, besides simply being below a certain threshold, reducing visit-to-visit fluctuations of blood pressure are equally important. Controlling the SD of systolic BP across visits below 7.5 is beneficial for delaying the progression of IgA nephropathy patients. Time-averaged systolic BP has been found to be independently associated with the need to start renal replacement therapy in patients with IgAN [10]. The impact of BP on IgAN is not simply linear, and in one study showed a U-shaped pattern [11]. In a retrospective cohort study included 1530 patients with IgAN, a U-shaped association between systolic BP and disease progression (ESRD or 30% decrease of eGFR) was found, especially in patients with proteinuria ≥ 1 g/day and eGFR ≥ 60 mL/min/1.73m^2^. Compared with systolic BP controlled at 110–119 mmHg, the hazard ratio (HR) for renal progression increased significantly from 1.48 at < 110 mmHg to 2.21 at 130–139 mmHg and 2.91 at ≥ 140 mmHg. Therefore, in patients with IgAN, the target BP is not necessarily lower but rather moderate, reduces fluctuations, and maintains long-term stability. Moreover, strict control of systolic BP may be more beneficial for patients with proteinuria greater than 1 g/d and early renal function impairment (CKD stages 1-3a). In a retrospective study involving 2240 patients with IgA nephropathy, compared to systolic BP < 120mmHg, systolic BP 120-139mmHg was a risk factor for disease progression only in patients with proteinuria > 1 g/d and CKD 1-3a stage, but not those with proteinuria ≥ 1 g/d and CKD 3b-4 stage [12].

Proteinuria is the most common risk factor for kidney disease, and when persistent, is the most powerful predictor of the prognosis [5]. In recent years, there has been significant progress in our understanding of the impact of proteinuria on IgAN. In 2012, Chinese researchers published the results of a 20-year follow-up study of 1155 patients with IgAN in which those with a urinary protein quantification value in the range of 0.5–1 g/day had a 10-year dialysis-free survival rate of 95% and a 20-year dialysis-free survival rate of 89% [13]. A study from 2020 found a non-linear relationship between duration of remission of proteinuria and the renal survival in 1864 patients with IgAN [14]. That study found that continuous improvement in the urine protein quantification value for more than 3 months and up to 4 years was associated with an additional 9% reduction in the risk of deterioration of renal function (composite of ESKD or a 50% decline in eGFR). That research suggested that there is no shortest duration of relief of proteinuria in patients with IgAN, and that even brief relief (3 months) is associated with a significant reduction in the risk of renal progression. However, each additional 3months beyond 4 years in remission was associated with a smaller, nonsignificant risk reduction. The possible reasons for the above results are that patients with longer remission duration beyond 4 years had a lower proteinuria, and were much more likely to achieve proteinuria < 0.3 g/d compared with those shorter remission duration. Subsequently, multiple studies have shown that time-averaged proteinuria is an independent risk factor for progression of IgAN [15, 16]. The 2021 KDIGO guidelines set a reduction in urine protein quantification of < 1 g/day as an alternative marker for improvement in the renal survival in these patients [5]. However, recent research has shown that even a urine protein quantification value of < 1 g/day has a significant impact on the long-term prognosis of IgAN [3, 17]. 30% of patients in the IgAN cohort from the UK National Registry of Rare Kidney Diseases study had an average proteinuria value of 0.44–0.88 g/g (equivalent to a urine protein quantification of 0.5–1 g/day) and 20% of those with an average value of < 0.44 g/g developed renal failure within 10 years [3]. Compared with time-varying proteinuria < 0.3 g/day, the respective HRs for proteinuria of 0.3 to < 0.5 g/day, 0.5 to < 1.0 g/day, 1.0 to < 2.0 g/day, and ≥ 2.0 g/day were reported to be 2.22 (95% confidence interval [CI] 0.88–5.58), 4.04 (95% CI 1.93–8.46), 8.46 (95% CI 3.80–18.83), and 38.00 (95% CI 17.62–81.95) [17]. Therefore, the composite kidney outcome starts to increase significantly when the time-averaged proteinuria is > 0.3 g/day.

The KDIGO guidelines indicate that a urinary protein quantification of > 1 g/day on maximum supportive care predicts progression of IgAN and requires immediate adjustment of the treatment regimen. Hematuria is not an indicator that requires treatment. In an experimental model of IgAN, hematuria was observed after deposition of IgA-IgG immune complexes (ICs), inflammation, and activation of the complement pathway. The oxidative damage caused by release of hemoglobin is believed to contribute to the onset and progression of proteinuria [18]. Furthermore, there is an increasing body of clinical evidence suggesting that hematuria may be a risk factor for progression of IgAN [18, 19]. The time-averaged hematuria threshold for predicting progression of the disease is 201 RBC/µL in women and 37 RBC/µL in men [20]. The author of that study suggests that the different thresholds between man and woman may be due to the structure of a woman’s urethra being different from man’s. But we speculate that the higher baseline urine RBC/uL in women compared to men may be another. A study from 2018 that included 988 patients with IgAN, both the degree of microscopic hematuria and the proportion of gross hematuria are significantly higher in females than in males [21]. A meta-analysis involving a total of 5660 IgAN patients from 13 studies also showed that initial microscopic hematuria was associated with an 87% increase in the risk of ESRD [22]. Moreover, in a multicenter retrospective study involving 2047 IgA nephropathy patients, it was found that hematuria was one of the 10 most important variables for a prediction model of IgAN progression [23]. One study found that resolution of hematuria can reduce the composite renal outcome of IgAN [19]. However, subgroup analysis in that study found that remission of hematuria had effect on progression of the disease only in the subgroup with persistent proteinuria, but not detectable within patients whose proteinuria had remitted. In the same study, the prognosis was significantly worse in patients with a time-averaged proteinuria of > 0.75 g/day when hematuria was persistent than in those in whom it was not. However, another study found no significant difference in the renal survival in patients with IgAN and time-averaged proteinuria of < 0.75 g/day according to whether or not hematuria was persistent [24]. Persistent hematuria often indicates increased IgAN activity. In a study that included 112 IgA nephropathy patients with an average follow-up of 14 years, patients with persistent hematuria (*n* = 46) presented a higher urinary protein quantification and M1 ratio than those with negative or minimal hematuria (*n* = 66) [24]. Moreover, patients with IgAN in whom hematuria persists after treatment are more likely to be male and to have a higher MAP, a higher proportion of M1, and glomerular segmental sclerosis or adhesion (S1) than those in whom hematuria resolves following treatment [19]. The proposed mechanism of the link between segmental sclerosis and haematuria may be related that RBCs release products such as hemoglobin and miRNAs, leading to oxidative damage, podocyte dysfunction, and eventually leads to disruption of the glomerular filtration barrier [20]. Although the relevant studies have used stratification or multivariate methods for analysis, it is difficult to exclude the influence of bias (selection bias and confounding bias) and confounding variables. Future research should control for bias using methods such as propensity score matching and target trial emulation [25] and use multicenter, large-sample prospective or retrospective cohort designs to simulate randomized controlled studies to the greatest extent possible in order to draw more reliable conclusions.

### Galactose-deficient IgA1

The first step in the widely recognized “four-hit hypothesis” of IgAN is to presume that the production of a large amount of galactose-deficient immunoglobulin A1 (Gd-IgA1) may be caused by mucosal immune stimulation [1]. Gd-IgA1 in serum and urine is considered to have diagnostic value for IgAN [26, 27] but whether it can predict progression of the disease is controversial. Several studies have found that high serum and urinary Gd-IgA1 levels before renal biopsy are associated with pathological focal sclerosis and renal tubulointerstitial fibrosis [28–30]. Patients with IgAN and renal tubulointerstitial fibrosis have a higher urinary Gd-IgA1 level [28], and the plasma Gd-IgA1 level is an independent predictor of renal tubular atrophy/interstitial fibrosis (the T grade) in these patients [31]. Multiple studies of IgAN from different eras, countries, and centers have found a close relationship between the Gd-IgA1 level in blood or urine and renal tubulointerstitial fibrosis [28–32]. However, when the 1982 World Health Organization grading system was used, there was no significant difference in the Gd-IgA1 level according to whether IgAN was mild or severe. Furthermore, recent meta-analyses found no clear evidence of a link between the Gd-IgA1 level and the progression of IgAN [33, 34]. A study that included 230 patients with IgAN who were followed up for an average of 22 months found that an elevated serum Gd-IgA1 level was an independent risk factor for progression of chronic kidney disease (CKD, namely, a decrease in eGFR of > 25% or a decrease in eGFR classification) [30]. However, that study did not include pathological classification. Another study from 2012 that included 275 patients with IgAN identified an elevated serum Gd-IgA1 level to be an independent risk factor for the composite endpoint of a decrease in eGFR of > 50%, ESRD, and death [35]. Even after incorporating progressive factors such as time-averaged proteinuria, hypertension, and eGFR, the serum Gd-IgA1 level could still predict progression of IgAN. However, that study was performed before the Oxford classification was adopted and used the Haas classification. A subsequent study of 946 patients with IgAN from 2019 also found an elevated serum Gd-IgA1 level to be an independent risk factor for the composite endpoint of a permanent reduction in eGFR of ≥ 40% from baseline, ESRD, and death [36]. Even after incorporating clinical data at the time of biopsy (age, sex, eGFR, proteinuria, MAP, renin-angiotensin inhibition status before biopsy, and use of immunosuppression therapy during follow-up) and the MEST-C score, the serum Gd-IgA1 level still predicted progression of IgAN. However, addition of the serum Gd-IgA1 level to a model containing clinical data and the MEST-C score did not improve the predictive performance further (C statistic, 0.79 [95% CI 0.73–0.84] vs. 0.80 [95% CI 0.73–0.85], *P* > 0.05). There is currently insufficient evidence to suggest that the Gd-IgA1 level is independently associated with the progression of IgAN. However, preliminary research in a small sample has suggested that multiple structural features of N-glycosylation and O-glycosylation of IgA are associated with progression of IgAN and that their predictive value is higher than that of the Gd-IgA1 level [37]. By using McFadden adjusted pseudo-R2, the structural features of N-glycosylation and O-glycosylation of IgA may be better predictors of IgAN than Gd-IgA1 level. Indeed, even if a third step, namely, mesangial deposition of Gd-IgA1-containing immune complexes, does occur, it may not necessarily cause clinical symptoms or positive laboratory results. Renal pathology performed as part of an autopsy study in patients without kidney disease often showed deposition of IgA in the mesangial area and pathological manifestations of IgAN, including proliferation of mesangial cells [38]. In another study, renal biopsy in healthy kidney donors before transplantation found IgA deposition in the mesangial area in 26% of cases in the absence of any clinical manifestations or symptoms [39].

### Urinary biomarkers

#### Proteins

As early as 2008, a study identified urinary epidermal growth factor (EGF), urinary monocyte chemoattractant protein-1 (MCP-1), and the urinary EGF/MCP-1 ratio to be independent risk factors for doubling of blood creatinine and/or ESRD in a median 54 [35–84] months in 132 patients with IgAN (Table 1) [40]. The investigators found that the urinary EGF/MCP-1 ratio had the highest area under the curve (AUC) for predicting progression of IgAN (0.91) and was closely associated with the slope of the decrease in estimated creatinine clearance. Another study found that the urinary EGF level predicted an annual decrease in creatinine clearance of more than 10% in 33 patients with IgAN [41]. There is still controversy regarding whether urinary kidney injury molecule-1 (KIM-1) predicts progression of IgAN. One study showed that it may be an independent risk factor for progression of IgAN to ESRD [42]. However, in the urine biomarker testing of IgAN patients in the “STOP-IgAN” [43], urinary KIM-1 did not appear to predict progression of IgAN or clinical remission [44]. As early as 1996, an Italian study that included 41 patients found that the urinary interleukin (IL)-6/EGF ratio could predict progression of IgAN within 3 years [45]. Another study from 2002 in which 59 Japanese patients with IgAN were followed for a median of 8 years found that the urinary IL-6 level was significantly higher in patients with disease progression than in those without progression [46]. In a study published in 2023 that included 762 patients with IgAN who were followed up for a median of 65 months, increased urinary IL-6 was again identified to be an independent risk factor for progression of IgAN (a > 50% decrease in eGFR or ESRD) [47]. Moreover, the C statistic of urinary IL-6 combined with clinical data (baseline eGFR, proteinuria, and hypertension) is as high as 0.84. Urinary IL-6 may have potential to replace pathological parameters (MEST-C) in models like the IIGANPT. Studies from various periods and countries have repeatedly identified elevated urinary IL-6 to be an independent risk factor for progression of IgAN. Meanwhile, there is no conclusive evidence to indicate that any other cytokines, such as urine neutrophil gelatinase-associated lipocalin (NGAL), urine tissue inhibitor of metalloproteinase-2, insulin-like growth factor binding protein 7, and urinary calprotectin, are biomarkers of progression of IgAN [44, 48].

**Table 1:** Urinary protein biomarkers for IgAN independent progression of clinical and/or pathological indicators

Biomarker	Nationality	Sample size	Progression definition	Follow up time	Adjustment for others factors (multivariate Cox’s regression)
Urine EGF, MCP-1, EGF/MCP-1 ratio [40]	Italy	132	doubling blood creatinine and/or ESRD	54 months	age, sex, eCrCl, proteinuria, histologic grade, and hypertension
Urine IL-6 [46]	Japan	59	Ccr of less than 60 ml/min	8.07 ± 1.72 years	age, sex, LEE grade, hypertension, and proteinuria
Urine IL-6 [47]	China	762	50% eGFR decline or ESRD	65 months	age, sex, eGFR, proteinuria, hypertension, Oxford classification scores, and treatment factors
Urinary C4d/creatinine [49]	China	508	50% eGFR decline or ESRD or death	36 months	sex, age, baseline proteinuria, hypertension, eGFR, MEST-C score, and immunosuppressive therapy
Fractional excess of IgG/surviving glomeruli ratio [50]	Italy	37 (Crescentic IgA Nephropathy)	ESRD and doubling of Scr	60 ± 40 months	Scr, FEIgG, FEα1m/SG, FEα1m, TID score, GGS, 24hP/SG, 24hP, and cellular crescents
Urinary IgG [51]	China	105 (IgAN with high proportion of global glomerulosclerosis)	eGFR of ≥ 50% or ESRD or death	37 (27.5–59) months	age, female, SBP, DBP, MAP, TP, TC, TG, LDL, IgG, IgGU, urinary RBC, Scr, UA, ALB, eGFR, glomerulosclerosis rate, Oxford classification, Immunosuppressive therapy, and RASIs therapy
Urinary C4d/creatinine [52]	China	168 (IgAN with crescentic lesions)	ESRD	19 months	age, sex, Scr, proteinuria, MAP, Oxford classification scores, and immunosuppressive therapy use
Urine periostin [60]	South Korea	399	ESRD	27.1 months	age, sex, urine protein-creatinine ratio, Scr
Urine CXCL1 [61]	China	425	50% eGFR decline, ESRD or death	47.46 ± 25.88 months	age, sex, proteinuria, eGFR, SBP, DBP, Oxford classification, RASIs therapy, and immunosuppressive therapy
Urine MMP-7, urine AGT, urine EGF, urine KIM-1, and serum Gd-IgA1 [36]	China	946 [the training set (*n* = 554) and the validation set (*n* = 392)]	permanent reduction ≥ 40% in eGFR over baseline, ESRD, or death	40 months in the training set and 28 months in the validation set	age, sex, eGFR, proteinuria, MAP, use of RAS inhibition before biopsy, use of immunosuppression during follow-up, and Oxford MEST-C score
237 urine peptides (IgAN237) [63]	Multinational cooperation	209	tertiles of the annual change of eGFR	39.4 ± 27.0 months	age, sex, eGFR, proteinuria, and MAP
sLG1M, sPRO-C3, sPRO-C6, uPRO-C6/Cr, uC3M/Cr [66]	Czech Republic	134	mean increase of Scr at least of 35%, and ESRD	56.5 months	eGFR, proteinuria, and Oxford classification

EGF: epidermal growth factor; MCP-1: monocyte chemoattractant protein-1; ESRD: end-stage renal disease; eCrCl: estimated creatinine clearance; IL-6: Interleukin 6; eGFR: estimated glomerular filtration rate; Ccr: creatinine clearance; LEE: Lee pathologic grading; Scr: serum creatinine; MAP: mean arterial blood pressure; FEIgG: the fractional excess of IgG; FEα1m/SG: the fractional excess of α 1-microglobulin/surviving glomeruli ratio; TID: tubulointerstitial damage; GGS: global glomerular sclerosis; 24hP/SG: 24 h protein surviving glomeruli; SBP: systolic blood pressure; DBP: diastolic blood pressure; TP: total protein; TC: total cholesterol; TG: triglyceride; LDL: low-density lipoprotein cholesterol; IgGU: urine immunoglobulin G; ALB: albumin; UA: uric acid; RASIs: renin-angiotensin-aldosterone system inhibitors; CXCL1: chemokine (C-X-C motif) ligand 1; MMP-7: matrix metalloproteinase 7; AGT: angiotensinogen; KIM-1: kidney injury molecule-1; Gd-IgA1: galactose-deficient immunoglobulin A1; sLG1M: serum fragment of MMP-laminin 1degragation; sPRO-C3: N-terminal pro-peptide of collagen type III in serum; sPRO-C6: C-terminal pro-peptide of collagen type VI in serum; uPRO-C6/Cr: C-terminal pro-peptide of collagen type VI measured in urine normalized by urine creatinine; uC3M/Cr: fragment of MMP-mediated degradation of collagen type III measured in urine normalized by urine creatinine

In addition to the more common cytokines and inflammatory factors, some complement and immunoglobulin-related proteins in urine may also predict progression of IgAN [49–51]. In a study of 508 patients with a urine protein quantification of > 1 g/day, an increase in the urinary C4d/creatinine ratio was an independent risk factor for progression (a > 50% decrease in eGFR, ESRD, or death) [49]. Higher urinary C4d predicted poor efficacy of treatment with glucocorticoids or immunosuppressants over a period of 3 months [49]. Furthermore, an increase in the urine C4d/creatinine ratio was identified to be an independent risk factor for progression to ESRD in patients who had IgAN with crescent formation [52]. The urinary C4d/creatinine ratio may be a useful predictor of progression of IgAN, and further research is warranted to determine whether it improves the predictive ability of the IIGANPT. The results of a study that included 37 patients with crescentic IgAN suggested that the fractional excess of the IgG/surviving glomeruli ratio was the strongest predictor of progression; however, when the serum creatinine level, fractional excess of α1-microglobulin, percentage of global glomerular sclerosis, 24-hour urinary protein, and other factors were included, this ratio was no longer an independent predictor of progression [50]. A study from 2022 identified the urine IgG level to be an independent predictor of the renal survival in 105 patients with IgAN and a high global sclerosis burden [51]. Another study found that the urine mannose-binding lectin level could distinguish between patients with and without complete remission of IgAN and was associated with both clinical indicators, including hypertension, proteinuria, blood creatinine, and eGFR, and pathological indicators, such as M, E, S, and T grade [53]. However, there was no significant correlation between the urine mannose binding lectin level and progression of IgAN in that study.

We have identified that the renal survival of IgAN is significantly poorer (a > 50% decrease in eGFR or progression to ESRD) in patients with increased expression of urine angiostatin [54]. Angiostatin, a 38-kDa plasminogen fragment, is a potent angiogenic inhibitor that blocks proliferation, induces apoptosis, prevents migration of endothelial cells, and disrupts capillary integrity [55]. Other studies have demonstrated that the urinary angiostatin to creatinine ratio is significantly correlated with CKD, Lee’s grade, and M and T grades and that the urine Dickkopf-3 (DKK-3) level is closely associated with contrast-induced nephropathy, AKI, and progression of CKD [56–58]. For the analysis of urine biomarkers in patients with IgAN in the “STOP-IgAN”, it was revealed a close relationship between an increase in the urinary DKK-3 level and a decrease in eGFR within 6 months [59]. Furthermore, renal function was more stable when the urinary DKK-3 level was stable or decreased. These findings suggested that urinary DKK-3 may be associated with the renal survival of IgAN in the short term. In a multicenter (two centers) prospective cohort study of 399 patients with IgAN, urine periostin (uPOSTN) was associated with ESRD, and/or eGFR decrease of 30%, and/or eGFR decrease of 50%. After adjusted for age, sex, urine protein-creatinine ratio, and Scr, uPOSTN was an independently associated with ESRD in IgAN patients [60]. A study that included 425 patients with IgAN identified urinary chemokine (C-X-C motif) ligand 1 (CXCL1) to be an independent prognostic factor in patients with IgAN (a > 50% decrease in eGFR, progression to ESRD, or death) [61]. In that study, inclusion of urinary CXCL1 in a prediction model containing clinical indicators (age, sex, 1 plus eGFR, natural log–transformed proteinuria, and systolic pressure) and pathological indicators (only T grade) significantly improved the ability to predict progression of IgAN. This means that the combination of urinary CXCL1 and some T-grade related biomarkers may replace renal biopsy and play a non-invasive role in predicting the progression of IgA nephropathy. A multicenter prospective study of urine biomarkers in 946 patients with IgAN found that urinary matrix metalloproteinase 7 (MMP-7), urinary angiotensinogen, urinary EGF, urinary KIM-1, and serum Gd-IgA1 levels independently predicted progression of the disease [36]. The predictive power was highest for urinary MMP-7. The addition of urinary MMP-7 level to the model containing clinical data at biopsy and MEST-C significantly improved the risk prediction of IgAN progression (C statistic, from 0.79 to 0.85). Moreover, the use of immunosuppressants and RAS inhibitors does not alter the results of subgroup analysis. These results indicated that urinary MMP-7 may replace Oxford classification in the IIGANPT model.

Multiple pathways and mechanisms are involved in progression of IgA, and the current prediction models include clinical, pathological, and therapeutic indicators [4, 62]. The ability of a single biomarker to predict progression of IgAN is limited. The value of a single biomarker is more likely to be reflected in the combination of multiple types of biomarkers, establishing new biomarker spectra, and achieving accurate prediction of progression of IgAN. The relevant research has explored the ability of combinations of multiple urinary biomarkers to predict progression of IgAN [63, 64]. Mass spectrometry is one of the main methods used to study biomarkers because it can detect almost all types of urinary proteins at low levels. A multicenter (six centers in Europe and one center in Canada) urine mass spectrometry study in 209 patients with IgAN (87% of patients received RAAS blockers, and 38% of patients received immunosuppressive agents) published in 2022 is an example of this type of research [63]. In that study, 237 urinary proteins (IgAN237) score was compared between patients with and without progression using liquid chromatography-tandem mass spectrometry (with internally validated). Addition of the IgAN237 score significantly improved the IgAN prediction model established by clinical indicators, increasing the AUC from 0.72 to 0.89. The AUC of 0.89 achieved by addition of the IgAN237 score does not require the inclusion of pathological indicators, indicating that the IgAN237 score may be a noninvasive predictor of disease progression and replace Oxford classification in the IIGANPT model. A more recent study also found that the IgAN237 score was associated with the slope of chronic eGFR decline over 6 months [65]. Other researchers have found that addition of a combination of five laminin and collagen degradation products (sLG1M, sPRO-C3, sPRO-C6, uPRO-C6/Cr, and u-C3M/Cr) can significantly improve the predictive ability of models based on clinical indicators or both clinical and pathological indicators [66]. In their study, a prediction model that included these five biomarkers, eGFR, and proteinuriahad higher AUC than that in both clinical and pathological indicators (eGFR, proteinuria, MEST pathological subtype, AUC = 0.856) for prediction of the progression of IgAN.

### Non-coding RNA biomarkers

In the human genome, apart from a small amount of messenger RNA that directly codes for proteins, more than 90% of RNA is non-coding RNA [67]. Although non-coding RNAs do not participate in translation of proteins, they play an important role in cell function and development of disease. At present, miRNAs are still the main urinary non-coding RNA biomarkers [68, 69]. There has been some research suggesting that certain circular RNAs contribute to production of IgA1 glycosylation abnormalities by regulating expression of miR-148b [70]. One study found that urinary exosomal miR-199a-3p predicted progression of IgAN with an AUC of 0.749 [71]. However, that study included only 55 cases, so this finding needs to be confirmed in larger multicenter studies. In another study that included 20 patients with IgAN who were followed for an average of 8 months, urinary exosomal miR-4639 and miR-210 expression levels were significantly higher in the group with progression than in the group without progression [72]. To date, the research on urinary exosomal miRNAs in IgAN has focused mainly on diagnostic biomarkers [73, 74], and studies that have investigated the ability of these miRNAs to predict progression of renal impairment have been preliminary and performed in small samples. The current method used for extraction of urinary exosomes is ultra-high-speed centrifugation. However, ultra-high-speed centrifuges are expensive and time-consuming to operate, making it difficult to meet the needs to process large numbers of samples and provide rapid results in clinical practice. Furthermore, relatively few urinary exosome extraction kits are available for research in IgAN [71]. Further studies are needed to confirm whether there are differences in the miRNA biomarker profiles extracted by these kits and those extracted by ultra-high-speed centrifugation.

The separation method used for measurement of miRNAs in urine sediment is relatively simple in comparison with that for urinary exosomal miRNAs. As early as 2010, Chinese researchers found a positive correlation between the miR-200b expression level in urinary sediment and the rate of decline in eGFR in patients with IgAN [75]. They also found significant correlations of miR-200b in urinary sediment and urine protein quantification values with eGFR. Our research group has conducted a series of experiments on miRNAs in urinary sediment and found that urinary miR-21 and miR-205 are associated with complete remission of IgAN [76]. Therefore, we have concluded that these biomarkers can be used to identify renal tubular atrophy/interstitial fibrosis. In 2023, our team found that the expression level of miR-16-5p in urinary sediment was significantly higher in a group with progression of IgAN than in a group without progression and could predict the E grade (proliferation of capillary cells) in the Oxford classification [69]. In addition, we also found that the expression level of urinary sediment miR-185-5p in the IgAN progression group was significantly higher than that in the non-progression group. miR-185-5p is mainly localized in the renal tubular epithelial cells and is closely associated with the degree of tubular atrophy/interstitial fibrosis [77]. We are now planning to combine multiple miRNAs (such as miR-16-5p and miR-185-5p) in urinary sediment from various pathologically related cell sources and assess their ability to predict progression of IgAN. Our ultimate aim is to replace the pathological grading in the IIGANPT with a noninvasive method based solely on clinical and therapeutic indicators and multiple biomarkers.

### Cell shedding

The main types of cells shed in urine are RBC, white blood cells (neutrophils, lymphocytes, and monocytes), transitional epithelial cells, squamous epithelial cells, renal tubular epithelial cells, and a small number of podocytes. In 2002, researchers in the US found a close relationship between the number of podocytes in urine and progression of IgAN and glomerulosclerosis [78]. Subsequent research suggested a positive correlation between the number of podocytes in urine and the urine protein quantification value in patients with IgAN [79–81]. The number of podocytes in urine is significantly higher in patients with IgAN who also have glomerular segmental sclerosis [79]. In recent years, use of single-cell sequencing technology for detection and classification of cells in the urine of patients with kidney disease has been found to have some value in the diagnosis of focal segmental glomerulosclerosis [82], membranous nephropathy [83], and lupus nephritis [84] and in prediction of the response to treatment. Testing the transcriptome of renal tubular epithelial cells in the urine of patients with AKI can reflect the extent of damage and repair processes. A large number of adaptive progenitor cells for repair of AKI have also been seen in urine [85]. There are currently no reports on use of single-cell sequencing in the urine of patients with IgAN. In the future, systematic and detailed detection of cells shed in the urine of these patients, especially those with rapid progression, will help to clarify the possible mechanisms of progression of IgAN and allow timely intervention without the need for repeated renal biopsies.

### Biomarkers in blood

#### Proteins

One study identified that increased expression of the tumor necrosis factor receptor (TNFR) in blood was closely associated with progression of IgAN (a ≥ 30% decrease in eGFR) and that its AUC was higher than that for urine protein quantification and eGFR (Table 2) [86]. A prospective cohort study that included 180 patients with IgAN found that fibroblast growth factor-23 (FGF23) was a risk factor for progression of IgAN and an independent predictor of the slope of eGFR decline [87]. In a study from 2023 in which 2511 patients were followed up for an average of 10 years, both the C-terminus of FGF23 and intact FGF23 had a non-linear relationship with development of CKD [88]. Only patients with expression levels in the top quartile showed a significant increase in the incidence of CKD. Whether this non-linear relationship also exists in IgAN is worthy of further investigation. Researchers in Finland found that plasma insulin levels and homeostasis model assessment of insulin resistance predicted the risk of progression of IgAN [89]. However, they did not report the number of patients with diabetes. The poor prognosis of insulin resistance cannot be excluded in their study because of superposition of the impact of diabetic nephropathy. Another study of 174 patients with IgAN identified high-sensitivity C-reactive protein, serum albumin, and the total white blood cell count to be associated with progression of the disease [90]. However, that study used a definition of progression that is not widely recognized and included patients who had not undergone renal biopsy for at least 5 years, so its conclusions are questionable. The findings of a further study that included 86 patients suggested that IL-7 is associated with renal tubular atrophy/interstitial fibrosis in IgAN and that low IL-7 expression heralds a poorer renal survival [91]. In that study, immunofluorescence showed that IL-7 is mainly localized in renal tubular epithelial cells and that overexpression of IL-7 alleviated transforming growth factor β1 (TGF-β1)-induced production of extracellular matrix through the mTOR1 pathway. Serum IL-18 has also been identified as an independent predictor of the risk of progression of IgAN (doubling of blood creatinine, ESRD, or death) [92]. Previous research has suggested close correlations of NGAL with early diagnosis of AKI [93] and deterioration of renal function in CKD [94]. NGAL belongs to the lipoprotein family and is mainly produced and secreted by renal tubular epithelial cells after stimulation. A study from 2015 that included 91 patients with IgAN showed that the plasma NGAL level was an independent predictor of the risk of progression to CKD3 or higher [95].

**Table 2:** Blood protein biomarkers for IgAN independent progression of clinical and/or pathological indicators

Biomarker	Nationality	Sample size	Progression definition	Follow up time	Adjustment for others factors (multivariate Cox’s regression)
sLG1M, sPRO-C3, sPRO-C6, uPRO-C6/Cr, uC3M/Cr [66]	Czech Republic	134	mean increase of Scr at least of 35%, and ESRD	56.5 months	eGFR, proteinuria, and Oxford classification
Serum TNFR1, and TNFR2 [86]	South Korea	347	eGFR ≥ 30% decline compared to baseline	26 months	age, sex, systolic BP, uPCR, and eGFR
Serum FGF23 [87]	Sweden	180	entering ESRD or ≥ 50% reduction in eGFR and entering ESRD or ≥ 25% reduction in eGFR by 10 years	52 (12–171) months	age, sex, serum albumin, calcium, phosphate, PTH, 25(OH) vitamin D, baseline albuminuria, baseline eGFR, MAP, BMI, and RASIs therapy
Serum IL-18 [92]	China	76	doubling of baseline Scr, ESRD, and death	58 months	age, smoking, blood pressure, albumin, lipids, CRP, hemoglobin, eGFR, TID scores
Plasma NGAL [95]	South Korea	91	CKD stage 3 or above	37.6 ± 19.9 months	age, sex, hypertension, RASIs therapy, gross hematuria, Haas classification, proteinuria, cholesterol, and LDL
Serum C3 [97]	South Korea	343	ESRD and a doubling of the baseline Scr	53.7 ± 30.1 months	age, sex, presence of gross hematuria, mean arterial blood pressure, eGFR, proteinuria, BMI, total cholesterol, serum albumin, urinary PCR, tubular atrophy/interstitial fibrosis, mesangial C3 deposition
Serum IgA/C3 ratio [98]	China	217	decline of eGFR or developing ESRD	36 months	sex, age, proteinuria ≥1 g/day, hypertension, eGFR < 60 mL/min/1.73m^2^, and Lee’s grade
Plasma FHR-1 [100]	United Kingdom	112	doubling of Scr	77.5 ± 76.4 months	Oxford classification, eGFR, proteinuria, C4d + immunofluorescence, and △CFHR3-CFHR1
Plasma FHR-5 [101]	China	1126	30% eGFR decline or ESRD	43.5 (24.0–79.0) months	proteinuria, eGFR, hypertension, Oxford classification, steroid or other immunosuppressive therapy
Serum C4 [102]	China	1356	≥ 50% reduction in eGFR or ESRD or death	48 ± 23 months	sex, age, history of hypertension, UPE, eGFR, serum UA, IgA, C3, treatment with RASIs, mesangial hypercellularity, segmental sclerosis, tubular atrophy/interstitial fibrosis, crescents, and ratios of global sclerosis
serum IgG [103]	China	455	ESRD or an irreversible 50% eGFR reduction	42.2 months	age, sex, SBP, DBP, hemoglobin, serum albumin, Scr, BUN, UA, TG, CHOL, serum IgA, IgM, C3, C4, eGFR, 24-hour urinary protein, and Oxford classification
Plasma copeptin [110]	Netherlands	59	doubling of Scr, ESRD or start of immunosuppressive therapy	5 years	sex, MAP, 24-hour urine protein, and eGFR
Serum MMP-7 [111]	China	244	ESRD and a 50% decline in eGFR	81.9 (46.3-116.6) months	sex, age, eGFR, proteinuria, hypertension, history of preceding infection, gross hematuria, Oxford-MEST lesion classification, and steroid use
CD14 gene-159 C polymorphism [112]	South Korea	216	doubling of Scr or ESRD	86 ± 51.1 months	age, sex, hypertension, proteinuria, and baseline renal function
Interferon gene score [114]	China	59	progressed to ESKD or with 30% eGFR decline	30.36 months	age, sex
CARD9 gene polymorphism [116]	China	986	ESRD or doubled Scr	26.94 (17.02–39.42) months	age, sex, hypertension, baseline 24 h urine protein, and eGFR
rs412852-G allele in CFH [117]	China	204 (IgAN patients with microangiopathy)	≥ 50% reduction in eGFR or ESRD or death	4.7 (2.4–7.4) years	age, sex, MAP, proteinuria, CKD stage, and Oxford classification
ST6GAL1 allele rs7634389 [118]	China	711	doubling of Scr or ESRD	26.9 (17.0–39.0) months	age, sex, hypertension, eGFR, and proteinuria

sLG1M: serum fragment of MMP-laminin 1degragation; sPRO-C3: N-terminal pro-peptide of collagen type III in serum; sPRO-C6: C-terminal pro-peptide of collagen type VI in serum; uPRO-C6/Cr: C-terminal pro-peptide of collagen type VI measured in urine normalized by urine creatinine; uC3M/Cr: (fragment of MMP-mediated degradation of collagen type III measured in urine normalized by urine creatinine; Scr: serum creatinine; ESRD: end-stage renal disease; eGFR: estimated glomerular filtration rate; TNFR1: TNF receptor 1; TNFR2: TNF receptor 2; BP: blood pressure; uPCR: urine protein-creatinine ratio; FGF23: fibroblast growth factor-23; PTH: parathyroid hormone; BMI: body mass index; MAP: mean arterial blood pressure; RASIs: renin-angiotensin-aldosterone system inhibitors; IL-18: interleukin-18; TID: tubulo-interstitial damage; NGAL: neutrophil gelatinase-associated lipocalin; LDL: low density lipoprotein; PCR: protein-to-creatinine ratio; FHR-1: factor H-related protein 1; △CFHR3-CFHR1: deletion of CFHR3 and CFHR1 genes; FHR-5: factor H-related protein 5; UPE: urinary protein excretion; SBP: systolic blood pressure; DBP: diastolic blood pressure; BUN: blood urea nitrogen; UA: uric acid; TG: triglyceride; CHOL: cholesterol; MMP-7: matrix metalloproteinase-7; CARD9: caspase recruitment domain family member 9; CFH: complement factor H; ST6GAL1: ST6 beta-galactosamide alpha-2,6-sialyltranferase1

IgAN is an autoimmune disease, and its onset and progression are closely related to the innate immune response, adaptive immune response, and complement system [1]. As far back as 1997, a study of 50 patients with IgAN suggested that activation of serum C3 was closely related to progression [96]. A subsequent study in a larger sample of patients with IgAN confirmed that a decrease in the serum C3 level is an independent risk factor for doubling of creatinine and ESRD [97]. However, a decrease in the serum C3 level is relatively unusual in patients with IgAN. Subsequent research that focused on serum IgA and C3 found that the serum IgA/C3 ratio may be a more accurate predictor of progression of IgAN (a > 50% decrease in eGFR or ESRD) than the serum C3 or IgA level alone. Furthermore, the AUC for the serum IgA/C3 ratio was reported to be significantly higher than that for serum IgA and serum C3 (0.742 vs. 0.699 and 0.628, respectively) [98]. Complement factor H-related protein 1 (FHR-1) may be involved in the regulatory function of factor H, a major negative regulator that interferes with activation of C3. A study of 112 patients with IgAN found that the plasma FHR-1 level and the ratio of FHR-1 to factor H were significantly higher in patients who progressed than in those who did not and that there was a significant negative correlation between the plasma FHR-1 level and eGFR [99]. However, the definition of progression of IgAN was very broad (i.e., an annual decrease in eGFR of 5 mL/min/1.73 m^2^, a > 50% decrease in eGFR, ESRD, pathological proliferation of endocapillaries, cellular or fibrous crescents, or immunosuppressive therapy) and no subgroup analysis of renal progression was included. Another cohort study from the same period defined progression of IgAN as doubling of serum creatinine and found that the plasma FHR-1 level but not serum FHR-5 was an independent predictor of the risk of progression [100]. However, a study of 1126 patients with IgAN in China identified the plasma FHR-5 level to be an independent predictor of progression (a > 30% decline in eGFR or ESRD) [101]. As with complement C3, complement C4 has also been suggested to be a biomarker for progression of IgAN. A cohort study of 1356 Chinese patients found that the serum C4 level independently predicted the risk of progression of IgAN (a > 50% decrease in eGFR, ESRD, or death) [102]. However, another report from China in the same year suggested that although serum IgG, IgM, and C4 predicted the risk of progression of IgAN (a > 50% decline in eGFR, ESRD, or death) in univariate analysis, only serum IgG remained after multivariate analysis [103]. In that study, serum C3 and C4 levels were not independent predicters of the risk of progression. Considering the inconsistent results across the various cohorts, higher-level evidence from multicenter, prospective cohort studies is needed to determine whether serum complement can predict the likelihood of progression of IgAN.

The Fc receptor for IgA (FcaRI, or CD89) is a type I glycoprotein receptor expressed on bone marrow cells that has high affinity for serum IgA, especially poly IgA [104]. Soluble CD89 (sCD89) is present in patients with IgAN and can bind to IgA to form an IgA-CD89 complex. As early as 2010, there was a report suggesting that the plasma sCD89 level was significantly higher in patients with progression of IgAN (doubling of blood creatinine or ESRD) than in those without progression [105]. However, a subsequent cohort study of 326 patients with IgAN from South Korea did not find any association between the sCD89-IgA complex and progression (a > 30% decrease in eGFR) [106]. Furthermore, use of recombinant CD89 to detect plasma levels of poly IgA found no association between poly IgA complexes and progression of IgAN [107]. Plasma sCD89, the plasma sCD89-IgA complex, and urine sCD89 may be specific diagnostic biomarkers for IgAN [108], but there is still insufficient evidence of their ability to predict progression of the disease. When the precursor of arginine vasopressin (AVP) splits, it produces equal amounts of peptide (copeptin) and AVP [109]. A cohort study of 59 patients with IgAN found that a higher plasma copeptin level was closely associated with the composite renal endpoint and various other endpoints, including doubling of serum creatinine, ESRD, and initiation of immunosuppressive therapy [110]. The ability of the plasma copeptin level to predict composite renal endpoints is higher than that of MAP, proteinuria, and eGFR [110]. A cohort study in China identified serum MMP-7 to be an independent predictor of the risk of progression of IgAN (a > 50% decline in eGFR or ESRD). After adding serum MMP-7 to clinical indicators, the AUC for prediction of progression was comparable to that of a prediction model using clinical and pathological indicators (Oxford classification, MEST), indicating that a combination of clinical indicators and serum MMP-7 could replace the pathological classification requiring renal biopsy [111]. The cohort studies from China confirmed that both urinary and serum MMP-7 can predict progression of IgAN and improve prediction based on clinical and pathological indicators [36, 111]. In the future, independent cohorts from various countries and ethnicities can be considered for further validation.

### Genomics

CD14 on the cell membrane is a component of cellular lipopolysaccharide signaling. It has been reported that 159 C polymorphism in the CD14 gene is associated with progression of IgAN (doubling of serum creatinine or ESRD) and may be involved in attenuating the inflammatory response to various adverse stimuli [112]. In a meta-analysis published in 2021, angiotensin-converting enzyme insertion/deletion gene polymorphisms was associated with IgAN progression [113]. A study that included 59 patients with IgAN identified interferon (IFN) gene score to be an independent progression factor (a > 30% decrease in eGFR, or progression to ESRD) in patients with IgAN after adjustments for sex and age [114]. A cohort study of 8529 Chinese patients found that a novel rare nonsynonymous risk variant in VEGFA was associated with the increased risk of disease progression in IgAN [115]. In addition, the rare VEGFA mutation could cause a conformational change and increase the binding affinity of VEGFA to its receptors. Caspase recruitment domain family member 9 (CARD9) was identified as a susceptibility gene for IgAN. The rs10747047-C and rs10870077-C alleles (single nucleotide polymorphisms within CARD9) were independently related to the poor prognosis of IgAN patients after adjustments for age, gender, hypertension, baseline 24 h urine protein, and eGFR [116]. Moreover, patients with the rs412852-G allele in complement factor H (CFH) become an independent risk factor for ESRD in patients with microangiopathic lesion [117]. It has also been reported that the ST6GAL1 allele (rs7634389) is a risk factor for doubling of serum creatinine or ESRD in patients with IgAN. Even after adjusting for age, sex, hypertension, eGFR, and proteinuria, the ST6GAL1 allele remains an independent risk factor [118].

### Non-coding RNA biomarkers

Decreased expression of core-1-β1,3 galactosyltransferase 1 (C1GALT1) in patients with IgAN may promote abnormal glycation of IgA1. miR-148b downregulates the expression of C1GALT1 and has some diagnostic value in IgAN [119]. Furthermore, let-7b downregulates N-acetylgalactosaminyltransferase 2 (GALNT2) and can play a role in diagnosis of IgAN [120]. An international multicenter retrospective cohort study showed that a combination of miR-148b and let-7b had significantly improved diagnostic value in IgAN [121]. However, the AUC for this combination was only 0.76–0.82. Nevertheless, the combination of miR-148b and let-7b has not only improved diagnostic value but also some ability to predict progression of IgAN [122]. It has predictive value for annual rates of decline in eGFR > 3 L/min/1.73 m^2^, a > 50% decline in eGFR, and ESRD. Plasma miR-29a also has some value in terms of predicting complete remission and progression of IgAN (a > 50% decline in eGFR or ESRD), with respective AUCs of 0.745 and 0.764 [123]. In a retrospective cohort study of 50 patients with IgAN, there was a significantly increased likelihood of deterioration in renal function in those with a low serum exosomal miR-192 level [124]. Moreover, during 2 years of follow-up, the incidence of progression of IgAN (a > 50% decrease in eGFR or ESRD) was significantly higher in the group with a low exosomal miR-192 level. In a retrospective cohort study of 30 patients with IgAN, shorter time to doubling of Scr was seen in high plasma miR-21-5p expression [125]. Plasma miR-29a has also been reported to be closely associated with the progression and treatment responsiveness of IgAN by Kaplan-Meier analysis [123]. However, due to the small number of endpoint events, multivariate cox regression analysis cannot be performed. Research on the ability of non-coding RNA biomarkers in urine and blood to predict progression of IgAN focuses mainly on miRNAs; there have only been a few small studies of non-coding RNA, which have usually focused on their diagnostic value. In future research on non-coding RNAs in IgAN, lncRNAs or circular RNAs can be appropriately introduced to clarify their role in disease progression and combined with existing miRNA biomarkers to improve their predictive accuracy.

### Conclusion and perspectives

Researchers have made some advances in the study of biomarkers of progression of IgAN. Some biomarkers that can be used for prediction of progression in various directions have been identified, including possible “four-hit” pathogenesis, complement-related pathways, novel protein biomarkers, non-coding RNAs, and specific cell types. Few biomarkers have been proven to improve the risk prediction model of combining clinical and pathological indicators, but only urinary IL-6, urinary CXCK1, urinary and serum MMP-7, IgAN237, a combination of five laminin and collagen degradation products (sLG1M, sPRO-C3, sPRO-C6, uPRO-C6/Cr, and u-C3M/Cr) may be the most promise biomarkers for replacing renal biopsy parameters in the IIGANPT. In the future, we should combine multiple biomarkers and use biomarker spectra to improve the efficiency of prediction. More in-depth research is needed to identify further mechanisms via which IgAN progresses and to provide more clues and support for identification of additional biomarkers.

## Data Availability

No datasets were generated or analysed during the current study.

## Abbreviations

BP: Blood pressure
IgAN: IgA nephropathy
ESRD: End-stage renal disease
KDIGO: Kidney Disease: Improving Global Outcomes
AKI: Acute kidney injury
ICs: Immune complexes
AUC: Area under the curve
lncRNA: Long non-coding RNA
MAP: Mean artistic pressure
SD: Standard deviation
RCT: Randomized controlled trial
HR: Hazard ratios
RaDaR: UK National Registry of Rare Kidney Diseases
Gd-IgA1: Galactose-deficient immunoglobulin A1
CKD: Chronic kidney disease
EGF: Epidermal growth factor
MCP-1: Monocyte chemoattractant protein-1
eCrCl: Estimated creatinine clearance
KIM-1: Kidney injury molecule-1
IL-6: Interleukin-6
NGAL: Neutrophil gelatinase associated lipoprotein
TIMP2 • IGFBP7: Tissue inhibitor of metalloproteinase-2 and insulin like growth factor binding protein 7
DKK-3: Dickkopf-3
uPOSTN: Urine periostin
CXCL1: Chemokine (C-X-C motif) ligand 1
MMP-7: Matrix metalloproteinase 7
AGT: Angiotensinogen
circRNA: circular RNA
FSGS: Focal segmental glomerulosclerosis
MN: Membranous nephropathy
LN: Lupus nephritis
TNFR: Tumor necrosis factor receptor
FGF23: Fibroblast growth factor-23
IL-7: Interleukin-7
TGF-β1: Transforming growth factor β1
IL-18: Interleukin-18
FHR-1: H related protein 1
fH: Factor H
FcaRI/CD89: Fc-α Receiver
sCD89: Soluble CD89
AVP: Arginine vasopressin
IFN: Interferon
VEGFA: Vascular endothelial growth factor A
CARD9: Caspase recruitment domain family member 9
CFH: Complement factor H
C1GALT1: Core-1-β1,3 galactosyltransferase 1
GALNT2: N-acetylgalactosaminyltransferase 2
